# Association between dietary pattern, atherogenic index of plasma, and cardiovascular disease risk factors amongst adults: A cross-sectional cohort-based study

**DOI:** 10.1371/journal.pone.0343023

**Published:** 2026-02-26

**Authors:** Mehrab Sayadi, Pouria Azami, Mohammad Javad Zibaeenezhad, Nasrin Motazedian, Fatemeh Khademian, Mohaddeseh Hasanzadeh, Fatemeh Zibaeenejad, Seyyed Saeed Mohammadi, Houri Mousavinezhad, Zahra Daneshvar, Nader Parsa, Iman Razeghian-Jahromi

**Affiliations:** 1 Cardiovascular Research Centre, Shiraz University of Medical Sciences, Shiraz, Iran; 2 Transplant Research Center, Shiraz University of Medical Sciences, Shiraz, Iran; 3 Division of Human Nutrition, Department of Agricultural, Food and Nutritional Science, University of Alberta, Edmonton, Alberta, Canada; 4 Nutritional Health Team (NHT), Shiraz, Iran; International University of Health and Welfare, School of Medicine, JAPAN

## Abstract

**Background and aims:**

This study aims to examine the association between dietary patterns, the atherogenic index of plasma (AIP), and cardiovascular risk factors in adults aged 40–70 years in southern Iran.

**Methods and results:**

Participants’ dietary patterns were assessed using a Food Frequency Questionnaire with 35 food groups. Dietary patterns were categorized using Principal Component Analysis into three groups: Vegan, Western, and Carbohydrate-based. AIP was calculated as the log (triglycerides/HDL-C), and cardiovascular risk was assessed using a standard prediction model. Participants were classified based on their adherence to dietary patterns, and their cardiovascular risk was assessed. A total of 1,675 participants were included (mean age 53.4 ± 8.3 years; 43.5% men). The high AIP group had higher ASCVD risk scores (7.0 ± 7.7) compared to the low (3.9 ± 5.5) and intermediate-risk groups (3.9 ± 5.1). High adherence to the vegan diet was associated with lower AIP (0.41 ± 0.22) compared to low adherence (0.46 ± 0.23), with an adjusted β of −0.047 (95% CI, −0.072 to −0.021; P < 0.001). In contrast, neither Western (high: 0.45 ± 0.23 vs. low: 0.44 ± 0.23) nor carbohydrate-rich diets (high: 0.45 ± 0.23 vs. low: 0.44 ± 0.22) showed significant associations with AIP.

**Conclusion:**

The findings suggest that high adherence to a vegan diet is beneficial for cardiovascular health, as evidenced by lower AIP, a marker of atherosclerosis risk. It highlights the potential role of dietary interventions in reducing cardiovascular risk, with a focus on plant-based diets for improving lipid profiles and heart health.

## Introduction

Cardiovascular diseases (CVDs) are the leading cause of mortality globally, accounting for a significant proportion of deaths and health-related complications [1]. Atherosclerosis, characterized by the buildup of fatty deposits in the arterial walls, is a key contributor to heart attacks, strokes, and other cardiovascular events. Despite significant advances in medical research and treatment, the prevalence of CVD continues to rise worldwide, with Iran being no exception [2]. Here, CVDs remain the most common cause of death and disability, contributing substantially to the national healthcare burden [3].

Among the major risk factors for CVD, certain modifiable factors, such as diet, smoking, and high blood cholesterol, play a central role in disease progression. High levels of triglycerides, low HDL cholesterol, and increased LDL cholesterol are key contributors to atherosclerosis and cardiovascular risk [4]. While genetic predispositions and non-modifiable factors like age and family history also influence CVD risk [5], diet represents one of the most significant modifiable risk factors [6,7]. Poor dietary habits, particularly those associated with high intake of processed foods, unhealthy fats, and refined carbohydrates, have been linked to an increased risk of cardiovascular events [8].

Dietary patterns, which reflect the overall quality and composition of an individual’s diet, provide a broader understanding of how nutrition influences health outcomes [9]. The Western dietary pattern, characterized by high consumption of red meat, refined grains, and sugary foods, has been consistently associated with higher cardiovascular risk [10]. Conversely, plant-based diets, such as the vegan diet, have been linked to lower rates of CVD, likely due to their higher content of fiber, antioxidants, and unsaturated fats [11]. However, the relationship between dietary patterns and CVD risk factors, particularly markers like the atherogenic index of plasma (AIP), remains an area of significant research interest. AIP, defined as the ratio of triglycerides to HDL cholesterol, is a reliable marker of lipid imbalance and a strong predictor of cardiovascular disease [12]. Elevated AIP reflects high triglycerides and low HDL cholesterol, both of which promote atherogenic plaque formation in arteries [13,14]. This lipid imbalance contributes to endothelial dysfunction and plaque rupture, increasing the risk of cardiovascular events [15].

Given the rising rates of cardiovascular diseases, coupled with the lack of specific research on the relationship between dietary patterns and lipid profiles in the region, this study aims to investigate the association between dietary patterns, AIP, and cardiovascular disease risk factors among adults aged 40–70 years. By analyzing how dietary habits influence lipid metabolism and cardiovascular risk in this population, this study seeks to provide valuable insights into the potential for dietary interventions to mitigate the burden of cardiovascular diseases. Furthermore, understanding these associations can help inform public health strategies aimed at improving diet quality and reducing cardiovascular risk in high-risk populations, particularly in regions where CVD continues to be a major health concern.

## Materials and methods

### Study design and population

This cross-sectional study was performed between 2021 and 2024 using data from the Shiraz Heart Study (SHS), a prospective cohort aimed at identifying predisposing factors for cardiovascular diseases, including coronary heart disease, cerebrovascular disease, and peripheral arterial disease, in the general population of Shiraz, Iran [16]. A summary of the SHS is as follows: the study targets adults aged 40–70 years in Shiraz, the capital city of Fars Province, and focuses on assessing cardiovascular risks over 10 years. The SHS collects comprehensive data through family physician clinics, including demographic, socio-economic, clinical, and lifestyle information. Participants were excluded from the study if they had a history of coronary artery disease, including coronary artery bypass surgery, balloon angioplasty, or percutaneous coronary intervention. Participants with significant coronary artery occlusion (at least 75% in one or more coronary arteries or 50% occlusion in the left main coronary artery) were also excluded. Additionally, individuals who were pregnant at the time of enrollment were excluded. Other exclusion criteria included individuals with chronic diseases such as pulmonary conditions, renal diseases, gastrointestinal disorders, rheumatologic diseases, or cancers. These conditions could affect cardiovascular risk factors or interfere with the study’s results. All participants gave written informed consent and were informed of the study’s purpose, procedures, and their right to withdraw at any time. The study was approved by the Ethics Committee of Shiraz University of Medical Sciences (IR.SUMS.MED.REC.1398.1308) and conducted in accordance with ethical guidelines, including the Declaration of Helsinki. We accessed medical records between 01 January 2025 and 31 May 2025. All data were de-identified before analysis, and investigators did not have access to personal identifiers after data extraction.

### Measurements

Habitual dietary intake was assessed using a validated 112-item semi-quantitative food frequency questionnaire (SFFQ) and was compared with 24-hour dietary recalls [17]. The SFFQ and the reference recalls were administered during the same data-collection period, with all 24-hour recalls completed within two weeks of the SFFQ. Each participant completed three non-consecutive 24-hour dietary recalls (two weekdays and one weekend day), all administered by trained dietitians. The SFFQ includes 11 food domains—dairy, fruits, vegetables, meat, legumes and cereals, fast foods, sweets, oils and fats, nuts, sauces, and soft drinks. Participants reported their consumption frequency across 10 categories, ranging from “seldom/never = 0” to “6 or more per day = 10.” Reported intake frequencies were converted to daily consumption and multiplied by standard portion sizes for analysis. Consumption of ultra-processed foods was estimated by classifying all SFFQ items according to the NOVA food processing system and summing the intake of items categorized as ultra-processed [18].

Atherogenic index of plasma (AIP) is calculated as the logarithm of the ratio of triglycerides (TG) to high-density lipoprotein cholesterol (HDL-C) (log10[triglycerides/HDL-C]). Based on the AIP values, participants were categorized into three groups: low risk (AIP ≤ 0.11), intermediate risk (0.11 < AIP ≤ 0.22), and high risk (AIP > 0.22) [12]. The ASCVD risk was determined using the Pooled Cohort Equations, which are a component of the ASCVD Risk Estimator developed by the American College of Cardiology (ACC) and the American Heart Association [19,20]. The 10-year risk score was calculated based on factors such as age, gender, race, total cholesterol and HDL cholesterol levels, systolic blood pressure, history of diabetes, hypertension treatment, and smoking status.

For the current analysis, the following covariates were assessed using standardized questionnaires: age, sex, smoking status, alcohol consumption, ethnicity, anxiety level, socioeconomic status, medical history, medications used, and family history of illness. Additionally, the following biochemical parameters were measured after a 12-hour fast: TG, total cholesterol [21], high-density lipoprotein cholesterol (HDL-C), low-density lipoprotein cholesterol (LDL-C), and fasting blood sugar (FBS). Diabetes mellitus (DM) was diagnosed based on any of the following criteria: two FBS measurements of 126 mg/dL or higher, a 2-hour postprandial blood sugar level greater than 200 mg/dL, or the use of medications to manage blood sugar levels. Participants were classified as having dyslipidemia if they met any of the following criteria: TC levels of 200 mg/dL or higher, TG of 150 mg/dL or higher, LDL-C levels above 130 mg/dL, HDL-C levels below 40 mg/dL for men or below 50 mg/dL for women, or if they were currently taking lipid-lowering medications due to a prior diagnosis of dyslipidemia. Blood pressure was measured twice in each arm after a 10-minute rest period, and the average values were used for analysis. Hypertension (HTN) was defined as meeting any of the following criteria: an average systolic blood pressure (SBP) of 140 mmHg or higher, an average diastolic blood pressure (DBP) of 90 mmHg or higher, or the use of antihypertensive medication. Anthropometric measurements, including height, weight, hip circumference (HC), waist circumference (WC), wrist circumference (WrC), and waist-to-hip ratio (WHR), were assessed directly by trained personnel. Body mass index (BMI) was calculated by dividing weight (in kilograms) by the square of height (in meters). Socioeconomic status was assessed using self-reported education level (low: primary or less; intermediate: secondary/high school; high: university/college) and monthly household income (low, intermediate, high based on national income tertiles).

### Statistical analysis

In this study, participants’ nutritional status was assessed using a Food Frequency Questionnaire (FFQ). To analyze the food consumption patterns, we used Exploratory Factor Analysis [9,22,23]. A total of 35 food groups were defined, which allowed us to derive dietary patterns. The number of factors was determined using Principal Component Analysis (PCA), with factors selected based on the scree plot and explained variance. Only factors with eigenvalues greater than 1 were retained. To enhance the interpretability of the factors, Varimax rotation was applied. From this analysis, three distinct dietary patterns emerged: vegan, western, and carbohydrate-based patterns, explaining 23% of the variance. The carbohydrate-based pattern was characterized by high positive factor loadings (>0.2) for refined grains (white bread, rice, pasta), potatoes, sweets and desserts, and sugar-sweetened beverages. For each participant, a score was calculated for each dietary pattern based on the factor loadings exceeding |0.2|, and these scores were then standardized. Participants were classified into three groups for each dietary pattern according to tertiles of the factor score. The first group, with the lowest adherence, was classified as (Low); the second group, with average adherence, was categorized as (Intermediate); and the third group, with the highest adherence, was classified as (High). Before analyzing the data, normality was assessed using skewness, kurtosis, Q-Q plots, and P-P plots. Outliers were identified and excluded, resulting in a final sample of 1,675 participants with complete micronutrient data.

Descriptive statistics were used to summarize baseline characteristics, with continuous variables expressed as means ± standard deviations (SD) and categorical variables as frequencies and percentages. One-way analysis of variance (ANOVA) was used to compare continuous variables across AIP categories (low, intermediate, high risk) and dietary adherence groups (low, intermediate, high). Post hoc pairwise tests were performed where appropriate. Differences in categorical variables were assessed using the chi-square test, and non-parametric data with the Kruskal–Wallis test. Associations between dietary patterns and AIP were examined using multiple linear regression, with AIP as the dependent variable and dietary adherence (low, intermediate, high; low = reference) as the independent variable, adjusting for age, sex, and smoking. Results are reported as regression coefficients (β) with 95% confidence intervals (CI). Mediation analysis was performed to assess whether HDL’s effect on ASCVD risk was mediated through AIP, estimating ACME, ADE, total effect, and proportion mediated with 95% CIs and p-values. Sensitivity analyses were conducted after excluding participants with hypertension, diabetes, or hyperlipidemia. Exploratory interaction terms were added to test for effect modification by age, sex, smoking, and BMI. SFFQ validity was evaluated by comparing energy and nutrient intakes with the averaged values of three 24-hour recalls, using correlation analysis, mean differences, and tertile cross-classification. Statistical significance was defined as two-sided P < 0.05. All analyses were performed using R (version 4.3.1) in RStudio (Posit, Boston, MA), with the stats package for regression and hypothesis testing, the mediation package for mediation analysis, and ggplot2 for data visualization.

## Results

### Baseline characteristics

A total of 1,675 participants were included in the study, with a mean age of 53.4 ± 8.3 years; 728 (43.5%) were men. Based on AIP, 85.6% of participants were classified as high risk for cardiovascular disease, 7.5% as moderate risk, and 6.9% as low risk. Baseline characteristics across AIP categories are presented in **Table 1**. Participants in the high-risk AIP group were older (53.6 ± 8.2 years) than those in the low- and intermediate-risk groups (P = 0.013). The prevalence of men was higher in the high-risk group (45.3%) compared with the low (37.4%) and intermediate (27.8%) groups (P < 0.001). Smoking was also more common in the high-risk group (21.9%) relative to the low (11.3%) and intermediate (11.9%) groups (P = 0.001). The prevalence of diabetes was higher in the high-risk group (20.4%) compared with the low (12.2%) and intermediate (15.1%) groups (P = 0.043), while hyperlipidemia was also more frequent (38.4% vs. 17.4% and 23.0%, respectively; P < 0.001). Anthropometric and metabolic measures were consistently less favorable among participants in the high-risk AIP group. They had higher BMI (28.1 ± 4.4), hip circumference (104.3 ± 8.4), wrist circumference (17.1 ± 1.4), WHR (0.95 ± 0.06), FBS (106.1 ± 30.2), triglycerides (158.4 ± 69.5), LDL-C (109.5 ± 28.1), SBP (124.2 ± 16.5), and DBP (80.5 ± 10.0) compared with the low- and intermediate-risk groups (all P < 0.05). In contrast, HDL-C was markedly lower in the high-risk group (46.2 ± 8.9) compared with low (62.3 ± 11.6) and intermediate (58.8 ± 9.0) groups (P < 0.001). No significant differences in AIP categories were observed across education or income levels (both P > 0.05). Thus, socioeconomic status did not show a meaningful association with AIP in this population.

**Table 1:** Comparison of baseline data and demographic characteristics across AIP status groups.

Variables	AIP Risk Group			P-value
	Low risk (n = 115, 6.9%)	Intermediate risk (n = 126,7.5%)	High risk (n = 1434, 85.6%)	
**Age**	51.91 ± 8.63	51.87 ± 8.06	53.63 ± 8.23	**0.013**
**Gender (male)**	43 (37.4)	35 (27.8)	650 (45.3)	**<0.001**
**Smoke**	13 (11.3)	15 (11.9)	314 (21.9)	**0.001**
**History of DM**	14 (12.2)	19 (15.1)	293 (20.4)	**0.043**
**History of HTN**	20 (17.4)	23 (18.3)	331 (23.1)	0.193
**History of HLP**	20 (17.4)	29 (23.0)	551 (38.4)	**<0.001**
**BMI**	26.74 ± 5.74	26.92 ± 4.67	28.08 4.42	**<0.001**
**HC**	102.96 ± 10.48	103.20 ± 8.94	104.28 ± 8.37	**<0.001**
**WC**	93.90 ± 13.46	94.32 ± 11.84	99.16 ± 10.48	0.131
**WrC**	16.57 ± 1.25	16.48 ± 1.28	17.08 ± 1.37	**<0.001**
**WHR**	0.91 ± 0.07	0.91 ± 0.07	0.95 ± 0.06	**<0.001**
**FBS**	96.97 ± 12.31	97.9 ± 17.45	106.06 ± 30.17	**<0.001**
**TG**	64.75 ± 15.75	85.70 ± 13.42	158.36 ± 69.49	**<0.001**
**TC**	182.46 ± 38.17	188.64 ± 35.18	191.58 ± 41.26	0.064
**HDL_C**	62.28 ± 11.55	58.78 ± 8.98	46.24 ± 8.87	**<0.001**
**LDL_C**	100.24 27.88	104.75 ± 25.55	109.45 ± 28.13	**0.001**
**SBP**	120.53 ± 15.09	120.49 ± 15.54	124.22 ± 16.48	**0.005**
**DBP**	76.92 ± 9.26	77.89 ± 10.25	80.48 ± 10.01	**<0.001**
**Education:**				
Low	56 (8.4%)	49 (7.3%)	563 (84.3%)	0.164
Intermediate	31 (5.5%)	50 (8.8%)	485 (85.7%)	
High	28 (6.5%)	27 (6.2%)	379 (87.3%)	
**Income:**				
Low	8 (4.5%)	10 (5.6%)	160 (89.9%)	0.448
Intermediate	96 (7.3%)	105 (7.9%)	1123 (84.8%)	
High	11 (6.5%)	11 (6.5%)	148 (87.1%)	
**ASCVD risk score**	3.92 ± 5.54	3.92 ± 5.13	7.00 ± 7.66	**<0.001**

Data were presented as mean ± SD or number (%) for continuous and categorical data respectively.

DM: Diabetes mellitus; HTN: Hypertension; HLP: Hyperlipoproteinemia; BMI: Body max index; HC: Hip circumference; WC: Waist circumference; WrC: Wrist circumference WHR: waist-hip ratio; FBS: Fasting blood sugar; TG: Triglyceride; TC: Total cholesterol; HDL_C: High-density lipoprotein cholesterol; LDL-C: Low-density lipoprotein cholesterol; SBP: Systolic blood pressure; DBP: Diastolic blood pressure; AIP: Atherogenic index of plasma; ASCVD: Atherosclerotic cardiovascular disease.

**Fig 1:**
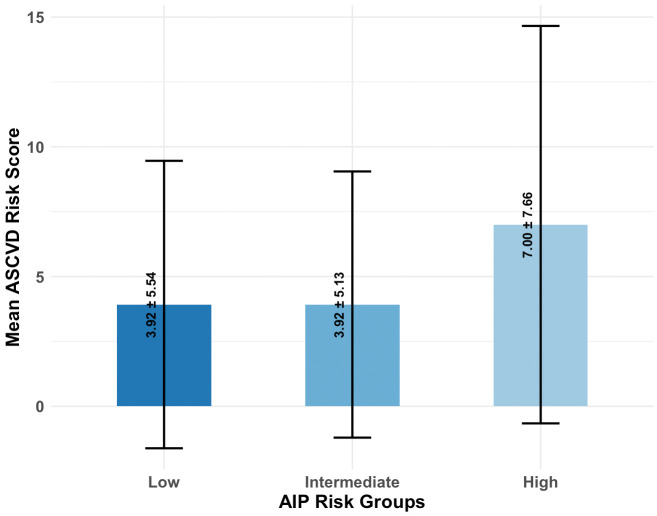
Mean ASCVD risk score (± SD) is shown for participants classified as low, intermediate, and high risk based on AIP. Participants in the high-risk AIP group had significantly higher ASCVD risk scores (7.0 ± 7.7) compared with the low (3.9 ± 5.5) and intermediate-risk (3.9 ± 5.1) groups (P < 0.001).

The ASCVD risk score differed across AIP categories, with the high-risk group showing higher scores (7.0 ± 7.7) than both the low- (3.9 ± 5.5) and intermediate- (3.9 ± 5.1) risk groups (both P < 0.001) (Fig 1). We performed a mediation analysis to investigate whether the effect of HDL on ASCVD risk operates through the AIP. The analysis revealed a significant indirect effect of HDL on ASCVD risk via AIP (ACME = −0.066, 95% CI: −0.091 to −0.041, p < 0.001), indicating that part of HDL’s protective effect is mediated by AIP. The direct effect of HDL not mediated by AIP was also significant (ADE = −0.094, 95% CI: −0.135 to −0.055, p < 0.001). Overall, the total effect of HDL on ASCVD risk was −0.16 (95% CI: −0.194 to −0.129, p < 0.001), with approximately 41% of the effect mediated through AIP (S1 Table). These findings suggest that AIP partially explains the relationship between HDL and ASCVD risk (S1 Fig).

### Validation of the SFFQ

To assess the validity of the SFFQ, energy and nutrient intakes obtained from the questionnaire were compared with the mean values derived from the three 24-hour dietary recalls. Moderate and statistically significant correlations were observed for energy (r = 0.41, p < 0.001), carbohydrates (r = 0.44, p < 0.001), protein (r = 0.47, p < 0.001), and total fat (r = 0.39, p < 0.001). Mean differences between the two methods were small and within acceptable ranges (energy: + 112 kcal/day; carbohydrates: + 8.6 g/day; protein: −3.2 g/day; fat: + 2.9 g/day). Cross-classification analysis showed that 72.4% of participants were classified into the same or adjacent tertile of intake, while only 6.8% were misclassified into opposite tertiles. These findings indicate acceptable agreement between the SFFQ and the 24-hour dietary recalls.

### Vegan dietary pattern

**Fig 2:**
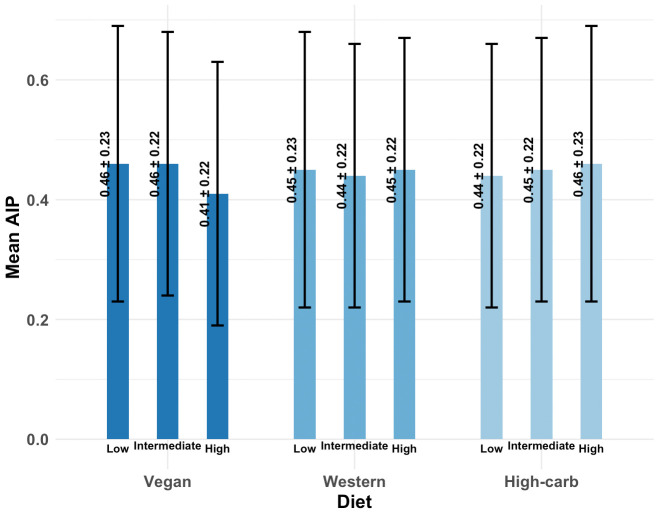
Mean AIP values across dietary pattern adherence categories. Unadjusted mean AIP (± SD) is shown for low, intermediate, and high adherence to vegan, Western, and high-carbohydrate dietary patterns. Participants with high vegan adherence had significantly lower AIP (0.41 ± 0.22) compared with low (0.46 ± 0.23) and intermediate (0.46 ± 0.22) adherence (P < 0.001). In contrast, AIP did not differ significantly across adherence levels to the Western or high-carbohydrate patterns.

Baseline characteristics according to adherence to the vegan dietary pattern are presented in **Table 2**. Participants with higher adherence were older (55.1 ± 8.1 years) compared with those in the low (51.5 ± 8.1 years) and intermediate-adherence (53.5 ± 8.2 years) groups (P < 0.001). The prevalence of hypertension decreased with increasing adherence (18.9% in the high-adherence group vs. 25.6% and 22.6% in the low- and intermediate-adherence groups, respectively; P = 0.026). Anthropometric and metabolic profiles were more favorable among those with greater adherence. Individuals in the high-adherence group had lower BMI (27.5 ± 4.8), waist circumference (97.5 ± 11.3), and triglyceride levels (139.3 ± 66.0), compared with the low- and intermediate-adherence groups (all P < 0.05). Conversely, HDL-C levels were higher in the high-adherence group (49.6 ± 10.7) compared with low (47.5 ± 9.6) and intermediate (47.8 ± 10.8) adherence (P = 0.002). Adherence to the vegan pattern varied significantly by socioeconomic indicators. Participants with higher education and higher income showed greater adherence to the vegan pattern (both P < 0.001). Importantly, AIP values were significantly lower in the high-adherence group (0.41 ± 0.22) compared with both low (0.46 ± 0.23) and intermediate (0.46 ± 0.22) groups (P < 0.001), consistent with the observed cardiometabolic benefits of this dietary pattern (**Fig 2**).

**Table 2:** Comparison of baseline data and demographic characteristics across different levels of adherence to the Vegan pattern.

Variables	Adherence to the Vegan pattern			P-value
	Low (n = 559)	Intermediate (n = 554)	High (n = 562)	
**Age**	51.49 ± 8.11	53.52 ± 8.21	55.12 ± 8.07	**<0.001**
**Gender (male)**	245 (43.8)	255 (46.0)	228 (40.6)	0.18
**Smoke**	106 (19.0)	119 (21.5)	117 (20.8)	0.557
**History of DM**	116 (20.8)	110 (19.9)	100 (17.8)	0.439
**History of HTN**	143 (25.6)	125 (22.6)	106 (18.9)	**0.026**
**History of HLP**	202 (36.1)	201 (36.3)	197 (35.1)	0.896
**BMI**	28.42 ± 4.61	27.85 ± 4.16	27.45 ± 4.83	**0.002**
**HC**	104.84 ± 8.78	103.97 ± 7.85	103.35 ± 9.02	**0.035**
**WC**	99.68 ± 11.1	98.13 ± 10.24	97.52 ± 11.30	**0.003**
**WrC**	17.16 ± 1.41	17.01 ± 1.33	16.82 ± 1.36	**<0.001**
**WHR**	0.95 ± 0.06	0.94 ± 0.06	0.94 ± 0.06	0.06
**FBS**	105.34 ± 26.38	105.34 ± 31.08	103.32 ± 28.34	0.298
**TG**	151.64 ± 80.31	148.46 ± 64.65	139.32 ± 66.01	**0.01**
**TC**	193.45 ± 40.23	189.58 ± 40.69	189.19 ± 41.04	0.153
**HDL_C**	47.50 ± 9.59	47.81 ± 10.76	49.55 ± 10.66	**0.002**
**LDL_C**	107.85 ± 27.58	107.92 ± 28.72	109.62 ± 27.79	0.492
**SBP**	125.25 ± 16.95	123.29 ± 16.24	122.51 ± 15.79	**0.015**
**DBP**	80.73 ± 10.51	80.03 ± 9.54	79.37 ± 10.00	0.077
**Education:**				
Low	305 (55.3%)	221 (39.5%)	142 (25.5%)	**<0.001**
Intermediate	146 (26.4%)	197 (35.2%)	223 (40.1%)	
High	101 (18.3%)	142 (25.4%)	191 (34.4%)	
**Income:**				
Low	85 (15.4%)	54 (9.6%)	39 (7.0%)	**<0.001**
Intermediate	428 (77.5%)	454 (80.8%)	442 (79.2%)	
High	39 (7.1%)	54 (9.6%)	77 (13.8%)	
**AIP**	0.46 ± 0.23	0.46 ± 0.22	0.41 ± 0.22	**<0.001**
**ASCVD risk score**	7.02 ± 7.61	6.66 ± 7.22	6.00 ± 7.51	0.067

Data were presented as mean ± SD or number (%) for continuous and categorical data respectively.

DM: Diabetes mellitus; HTN: Hypertension; HLP: Hyperlipoproteinemia; BMI: Body max index; HC: Hip circumference; WC: Waist circumference; WrC: Wrist circumference WHR: waist-hip ratio; FBS: Fasting blood sugar; TG: Triglyceride; TC: Total cholesterol; HDL_C: High-density lipoprotein cholesterol; LDL-C: Low-density lipoprotein cholesterol; SBP: Systolic blood pressure; DBP: Diastolic blood pressure; AIP: Atherogenic index of plasma; ASCVD: Atherosclerotic cardiovascular disease.

### Western dietary pattern

Baseline characteristics by adherence to the Western dietary pattern are shown in **Table 3**. Participants with low adherence were slightly older (53.9 ± 8.0 years) than those in the intermediate (53.1 ± 8.4 years) and high (52.7 ± 8.4 years) adherence groups (P = 0.038). The prevalence of smoking was highest among individuals with low adherence (26.1%) compared with the intermediate (16.9%) and high (18.3%) groups (P < 0.001). In contrast, cardiometabolic risk factors were more prevalent in the high adherence group. The proportions of diabetes (26.4%) and hypertension (25.6%) were highest in this group compared with the low- and intermediate-adherence groups (P < 0.001 and P = 0.032, respectively). Similarly, high adherence was associated with higher BMI (28.3 ± 4.7), waist circumference (99.5 ± 11.2), and wrist circumference (17.2 ± 1.4), along with less favorable lipid and metabolic profiles, including higher LDL-C (110.9 ± 27.6) and fasting blood glucose (107.6 ± 29.2) (all P < 0.05). Western diet adherence also differed significantly by socioeconomic status. Individuals with lower educational attainment and lower income were more likely to have higher adherence to the Western dietary pattern (both P < 0.001). ASCVD risk scores differed across Western diet adherence groups (P = 0.040), being higher in the high adherence group (7.1 ± 8.1) than in the low group (6.1 ± 6.9; P = 0.035), while the intermediate group (6.4 ± 7.0) did not differ significantly from either. Notably, unlike the Vegan dietary pattern, no significant differences were observed in AIP across the three Western diet adherence categories (P = 0.436).

**Table 3:** Comparison of baseline data and demographic characteristics across different levels of adherence to the western pattern.

Variables	Adherence to the Western pattern			P-value
	Low (n = 561)	Intermediate (n = 554)	High (n = 560)	
**Age**	53.94 ± 8.04	53.05 ± 8.35	52.70 ± 8.36	**0.038**
**Gender (male)**	304 (54.7)	242 (43.1)	182 (32.7)	**<0.001**
**Smoke**	145 (26.1)	95 (16.9)	102 (18.3)	**<0.001**
**History of DM**	78 (14.0)	101 (18.0)	147 (26.4)	**<0.001**
**History of HTN**	104 (18.7)	129 (23.2)	141 (25.6)	**0.032**
**History of HLP**	178 (32.0)	201 (35.8)	221 (39.7)	**0.029**
**BMI**	27.65 ± 4.67	27.73 ± 4.31	28.34 ± 4.66	**0.023**
**HC**	103.79 ± 8.25	104.06 ± 8.58	104.49 ± 8.90	0.388
**WC**	97.84 ± 11.05	98.44 ± 10.59	99.50 ± 11.18	**0.019**
**WrC**	16.88 ± 1.32	16.93 ± 1.38	17.17 ± 1.40	**0.001**
**WHR**	0.93 ± 0.06	0.94 ± 0.06	0.95 ± 0.06	**0.007**
**FBS**	103.88 ± 29.61	103.02 ± 26.97	107.59 ± 29.20	**0.018**
**TG**	147.82 ± 70.40	143.81 ± 70.64	146.98 ± 67.65	0.604
**TC**	189.99 ± 42.63	189.21 ± 42.63	193.00 ± 39.83	0.132
**HDL_C**	47.99 ± 10.40	48.40 ± 10.02	48.47 ± 10.72	0.703
**LDL_C**	107.29 ± 28.94	107.25 ± 27.44	110.89 ± 27.56	**0.046**
**SBP**	122.20 ± 16.80	124.22 ± 15.97	124.56 ± 16.20	**0.034**
**DBP**	80.07 ± 10.40	80.17 ± 9.94	79.88 ± 9.78	0.888
**Education:**				
Low	138 (25.1%)	206 (36.9%)	323 (57.7%)	**<0.001**
Intermediate	210 (38.3%)	200 (35.8%)	156 (27.9%)	
High	201 (36.6%)	152 (27.2%)	81 (14.5%)	
**Income:**				
Low	51 (9.3%)	54 (9.6%)	73 (13.1%)	**<0.001**
Intermediate	407 (73.9%)	457 (81.5%)	459 (82.1%)	
High	93 (16.9%)	50 (8.9%)	27 (4.8%)	
**AIP**	0.45 ± 0.23	0.44 ± 0.22	0.45 ± 0.22	0.436
**ASCVD risk score**	6.05 ± 6.85	6.39 ± 7.02	7.14 ± 8.08	**0.04**

Data were presented as mean ± SD or number (%) for continuous and categorical data respectively.

DM: Diabetes mellitus; HTN: Hypertension; HLP: Hyperlipoproteinemia; BMI: Body max index; HC: Hip circumference; WC: Waist circumference; WrC: Wrist circumference WHR: waist-hip ratio; FBS: Fasting blood sugar; TG: Triglyceride; TC: Total cholesterol; HDL_C: High-density lipoprotein cholesterol; LDL-C: Low-density lipoprotein cholesterol; SBP: Systolic blood pressure; DBP: Diastolic blood pressure; AIP: Atherogenic index of plasma; ASCVD: Atherosclerotic cardiovascular disease.

### High-carbohydrate dietary pattern

Baseline characteristics by adherence to the high-carbohydrate dietary pattern are presented in **Table 4**. Participants in the high adherence group were older (55.0 ± 8.1 years) compared with those in the intermediate (53.2 ± 8.2 years) and low (51.9 ± 8.2 years) groups (P < 0.001). Men were disproportionately represented in the high adherence group (66.9%) compared with the low (43.2%) and intermediate (59.3%) groups (P < 0.001). Similarly, smoking prevalence was substantially higher among those with high adherence (31.1%) compared with the low (11.8%) and intermediate (18.5%) groups (P < 0.001). High adherence was also associated with greater cardiometabolic burden. The prevalence of diabetes (24.5%) and hypertension (24.9%) was significantly higher in the high adherence group (P < 0.001 and P = 0.049, respectively), as was hyperlipidemia (42.6% vs. 26.9% in low adherence; P < 0.001). Participants in the high adherence group also had higher BMI (28.2 ± 4.4), hip circumference (104.8 ± 8.6), and wrist circumference (17.2 ± 1.3) compared to the other groups (all P < 0.01). In terms of lipid and metabolic profiles, LDL-C (110.5 ± 28.0) and total cholesterol (194.7 ± 41.3) were higher among high adherence participants (P < 0.01 for both), whereas HDL-C was lower (46.6 ± 9.6) compared with the low- and intermediate-adherence groups (P < 0.001). FBS, TG, and blood pressure did not differ among the high-carb adherence groups (P > 0.05). Participants with higher education and higher income were more frequently classified in the high-adherence group (P < 0.001). Neither ASCVD risk score (P = 0.801) nor AIP (P = 0.083) differed significantly across these groups.

**Table 4:** Comparison of baseline data and demographic characteristics across different levels of adherence to the high carbohydrate pattern.

Variables	Adherence to the high-carbohydrate pattern			P-value
	Low (n = 553)	Intermediate (n = 563)	High (n = 559)	
**Age**	51.91 ± 8.20	53.24 ± 8.18	54.98 ± 8.13	**<0.001**
**Gender (male)**	239 (43.2)	334 (59.3)	374 (66.9)	**<0.001**
**Smoke**	66 (11.8)	104 (18.5)	172 (31.1)	**<0.001**
**History of DM**	71 (12.8)	118 (21.0)	137 (24.5)	**<0.001**
**History of HTN**	103 (18.7)	128 (22.7)	139 (24.9)	**0.049**
**History of HLP**	149 (26.9)	213 (37.8)	238 (42.6)	**<0.001**
**BMI**	27.36 ± 4.57	28.12 ± 4.67	28.22 ± 4.39	**0.003**
**HC**	102.98 ± 8.12	104.45 ± 8.85	104.81 ± 8.64	**0.001**
**WC**	97.54 ± 11.09	98.90 ± 10.96	98.81 ± 10.77	0.059
**WrC**	16.83 ± 1.37	16.94 ± 1.40	17.23 ± 1.31	**<0.001**
**WHR**	0.94 ± 0.06	0.94 ± 0.06	0.95 ± 0.07	0.556
**FBS**	103.31 ± 23.22	104.60 ± 29.78	405.60 ± 27.84	0.369
**TG**	143.51 ± 68.63	148.50 ± 68.0	146.32 ± 72.11	0.476
**TC**	187.11 ± 40.76	190.44 ± 39.58	194.67 ± 41.27	**0.008**
**HDL-C**	49.69 ± 10.77	48.60 ± 10.49	46.56 ± 9.61	**<0.001**
**LDL-C**	105.26 ± 28.40	109.62 ± 27.44	110.52 ± 27.99	**0.004**
**SBP**	123.66 ± 16.68	123.53 ± 16.59	123.86 ± 15.84	0.944
**DBP**	79.31 ± 10.52	80.30 ± 9.51	80.60 ± 9.91	0.051
**Education:**				
Low	305 (55.3%)	221 (39.5%)	142 (25.5%)	**<0.001**
Intermediate	146 (26.4%)	197 (35.2%)	223 (40.1%)	
High	101 (18.3%)	142 (25.4%)	191 (34.4%)	
**Income:**				
Low	85 (15.4%)	54 (9.6%)	39 (7.0%)	**<0.001**
Intermediate	428 (77.5%)	454 (80.8%)	442 (79.2%)	
High	39 (7.1%)	54 (9.6%)	77 (13.8%)	
**AIP**	0.44 ± 0.22	0.45 ± 0.22	0.46 ± 0.23	0.083
**ASCVD risk score**	6.44 ± 7.28	6.56 ± 7.62	6.69 ± 7.47	0.801

Data were presented as mean ± SD or number (%) for continuous and categorical data respectively.

DM: Diabetes mellitus; HTN: Hypertension; HLP: Hyperlipoproteinemia; BMI: Body max index; HC: Hip circumference; WC: Waist circumference; WrC: Wrist circumference WHR: waist-hip ratio; FBS: Fasting blood sugar; TG: Triglyceride; TC: Total cholesterol; HDL_C: High-density lipoprotein cholesterol; LDL-C: Low-density lipoprotein cholesterol; SBP: Systolic blood pressure; DBP: Diastolic blood pressure; AIP: Atherogenic index of plasma; ASCVD: Atherosclerotic cardiovascular disease.

### Association of dietary patterns with AIP

We modeled AIP as a function of each dietary pattern (three adherence categories; low as reference), adjusting for age, sex, and smoking. As shown in **Table 5**, higher adherence to the vegan pattern was associated with lower AIP: high vs low β = −0.0466 (SE 0.0130; 95% CI −0.0721, −0.0211; P < 0.001). Intermediate adherence did not differ significantly from low adherence (β = −0.0041; P = 0.757). In contrast, adherence to the Western pattern showed no significant association with AIP (high vs low: β = 0.0216; 95% CI −0.0059, 0.0490; P = 0.123; intermediate vs low: β = −0.0017; P = 0.899), and adherence to the high-carbohydrate pattern was likewise not associated with AIP (high vs low: β = −0.0016; P = 0.910; intermediate vs low: β = 0.0115; P = 0.399). Unadjusted AIP means followed the same pattern (e.g., vegan high 0.41 ± 0.22 vs low 0.46 ± 0.23).

**Table 5:** Association of Vegan dietary pattern adherence with AIP in the total population and sensitivity subgroups.

Population Group (n)	Diet	Comparison (Adherence)	β (Estimate)	SE	95% CI	p-value
**Total (1675)**	Vegan	High vs Low	−0.04657	0.0130	**[−0.0721, −0.0211]**	**<0.001**
**No HTN (1304)**	Vegan	High vs Low	−0.04103	0.0153	**[−0.0710, −0.0110]**	**0.007**
**No DM (1349)**	Vegan	High vs Low	−0.05354	0.0149	**[−0.0827, −0.0243]**	**<0.001**
**No HLP (1075)**	Vegan	High vs Low	−0.05722	0.0164	**[−0.0894, −0.0251]**	**<0.001**

Data are presented as β coefficients with standard errors (SE) and 95% confidence intervals (CI) from adjusted regression models.

### Sensitivity analyses

**Fig 3:**
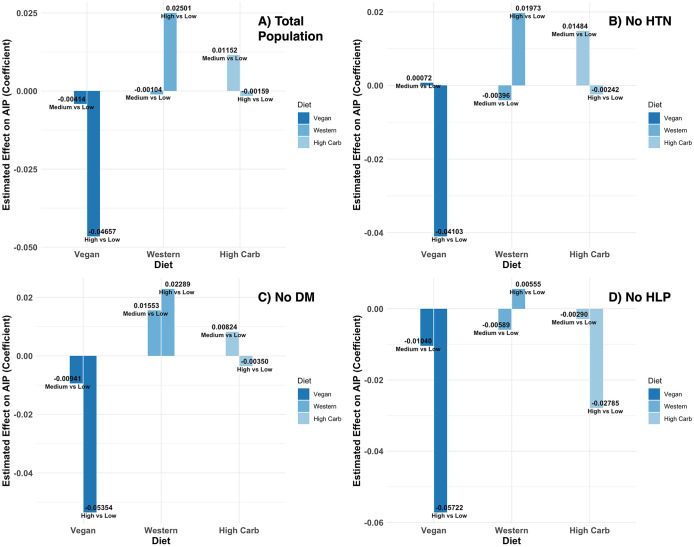
Associations of dietary patterns with the atherogenic index of plasma (AIP). Adjusted β coefficient for medium vs low and high vs low adherence to vegan, Western, and high-carbohydrate patterns are shown for the total population (A) and after excluding participants with hypertension (B), diabetes (C), or hyperlipidemia (D). Higher vegan adherence was consistently associated with lower AIP, while Western and high-carbohydrate patterns showed no significant associations.

To evaluate robustness, we repeated the adjusted models after excluding participants with hypertension, diabetes, or hyperlipidemia (see Table 5 and S2 Table). In the subgroup without hypertension (n = 1,304), high versus low vegan adherence remained inversely associated with AIP (β = −0.0410; 95% CI −0.0710, −0.0110; P = 0.007), whereas intermediate versus low adherence was null (P = 0.963). Western and high-carbohydrate patterns were not associated with AIP (all P ≥ 0.21). In the subgroup without diabetes (n = 1,349), the inverse association for high versus low vegan adherence was again present and slightly stronger (β = −0.0535; 95% CI −0.0827, −0.0243; P < 0.001), while intermediate versus low adherence was not associated (P = 0.532). Western and high-carbohydrate patterns remained null (all P ≥ 0.14). In the subgroup without hyperlipidemia (n = 1,075), high versus low vegan adherence remained significant (β = −0.0572; 95% CI −0.0894, −0.0251; P < 0.001), with no association for intermediate versus low adherence (P = 0.529). Western and high-carbohydrate patterns again showed no association with AIP (all P ≥ 0.72). Across all three sensitivity sets, results were directionally consistent with the primary analysis: higher vegan adherence was associated with lower AIP, whereas Western and high-carbohydrate adherence showed no statistically significant associations (Fig 3).

### Exploratory interaction analyses

**Fig 4:**
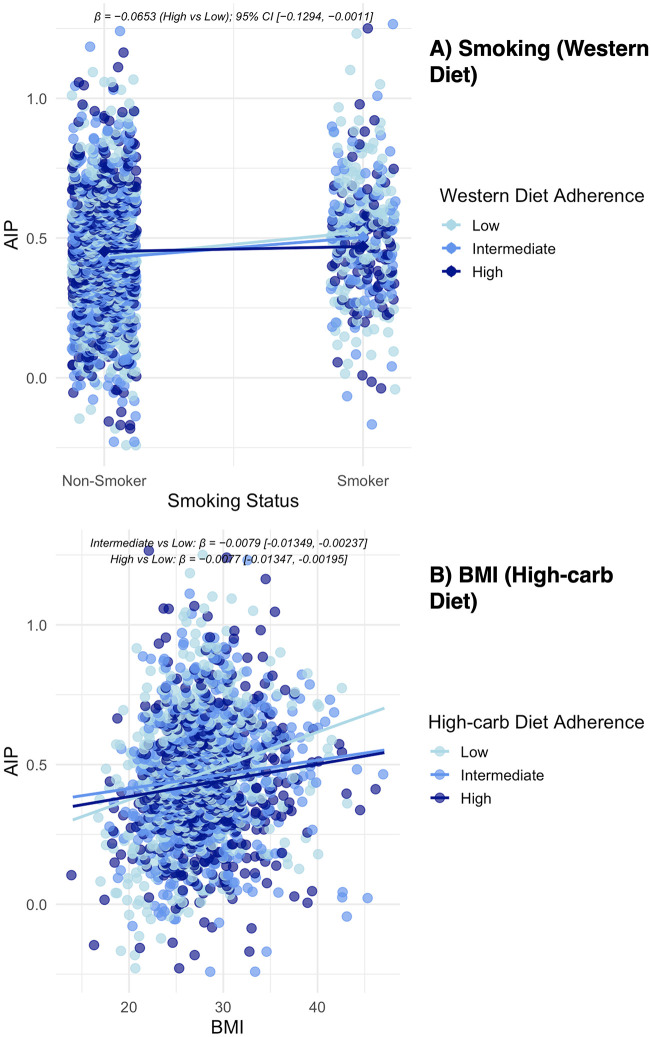
Associations of AIP with (A) smoking status across Western diet adherence and (B) BMI across high-carbohydrate diet adherence. Points represent individual observations, and lines represent regression-predicted trends adjusted for covariates. Interaction estimates (β, 95% CI) are shown above each panel.

We explored effect modification by age, sex, smoking, and BMI for each dietary pattern in models that included the corresponding main effects. Most interactions were not significant. For the Western pattern, the smoking × adherence interaction for high versus low adherence was negative and borderline significant (β = −0.0653; 95% CI −0.1294, −0.0011; P = 0.046), suggesting that the Western–AIP contrast was smaller among smokers than non-smokers. For the high-carbohydrate pattern, BMI × adherence interactions indicated that the slope of AIP versus BMI was weaker at intermediate and high adherence compared with low (intermediate vs low: β = −0.0079, P = 0.005; high vs low: β = −0.0077, P = 0.009) (Fig 4). Full coefficients and CIs are presented in S3 Table, but these findings should be interpreted cautiously.

## Discussion

This study aimed to investigate the association between dietary patterns, the AIP, and cardiovascular disease (CVD) risk factors among adults aged 40–70 years in southern Iran. The findings revealed that a significant majority (85.6%) of participants had high AIP levels, indicating an increased risk of CVD. The notably high proportion of individuals classified as high risk may reflect the age range of our sample, among whom atherogenic dyslipidemia is more common, and regional dietary and lifestyle patterns associated with elevated triglycerides and reduced HDL-C [24]. Given that the AIP cutoff used (AIP > 0.22) is a sensitive marker, this distribution is consistent with the high metabolic burden previously reported in this population. Individuals in the high AIP group were more likely to be older, male, smokers, and have higher rates of DM, HLP, obesity markers (BMI, WC, HC), elevated LDL-C and TG, lower HDL-C levels, and higher ASCVD risk scores. The analysis of dietary patterns showed that high adherence to a vegan diet was associated with lower BMI, blood pressure, and triglycerides, along with higher HDL-C levels, suggesting potential cardiovascular benefits. In contrast, high adherence to a Western diet was linked to higher rates of DM, HTN, HLP, LDL-C, fasting blood sugar, and a greater ASCVD risk score, indicating an increased risk for CVD. Similarly, a high-carbohydrate diet was associated with higher BMI, TC, LDL-C, and smoking prevalence, although HDL-C was also higher in this group. Regression analyses further showed that only high adherence to the vegan diet was independently associated with lower AIP, an effect that remained robust across sensitivity analyses, whereas Western and high-carbohydrate patterns showed no significant associations. Together, these findings underscore the potential of specific dietary patterns, particularly plant-based diets, in improving lipid profiles and reducing cardiovascular risk.

The analysis revealed significant differences in cardiovascular risk factors across the different AIP categories, with the high-risk AIP group showing a stronger association with metabolic and cardiovascular conditions. Participants in this group were more likely to have a history of diabetes, hypertension, and hyperlipidemia, which are well-established risk factors for atherosclerosis [25]. This suggests that higher AIP levels may reflect a metabolic environment predisposing individuals to these chronic conditions. Furthermore, the high-risk group exhibited higher BMI, WC, and waist-to-hip ratio, all of which are indicators of central obesity [26]. Central obesity is a key driver of insulin resistance and increased triglycerides, both of which contribute to higher cardiovascular risk [27–29]. The significantly higher fasting blood sugar and triglyceride levels seen in this group, alongside lower HDL-C and higher LDL-C, further support this pro-atherogenic profile, which is commonly linked to a higher risk of heart disease [30]. Additionally, the markedly higher ASCVD risk score in the high-risk AIP group reflects the cumulative effect of these metabolic abnormalities. A similar study conducted in Mexican women found that AIP was strongly correlated with several serum markers related to lipid metabolism (such as FABP4) and vascular health (such as ADMA), suggesting that AIP could serve as a valuable and affordable biomarker for early cardiovascular risk assessment [31]. Another study undertaken on 14,063 American adults found that higher AIP levels were significantly associated with an increased risk of diabetes mortality, particularly in women over 65 years old [32]. Given the associations between elevated AIP, poor metabolic health, and increased mortality risks, these findings emphasize the need for early monitoring of AIP levels and timely interventions to manage cardiovascular risk, particularly in high-risk populations such as older adults [25].

In the present study, participants with high adherence to the vegan pattern had a lower AIP score compared to those with intermediate and low adherence; notably, this association remained significant even after excluding individuals with HTN, DM, and HLP, supporting its robustness beyond potential confounding by pre-existing disease or medication use. Several mechanisms may explain the lower AIP observed among participants with high adherence to a vegan dietary pattern. Plant-based diets are rich in soluble fiber, which reduces hepatic triglyceride synthesis by slowing intestinal fat absorption and increasing bile acid excretion. High intake of unsaturated fatty acids enhances HDL-mediated reverse cholesterol transport, thereby lowering the TG/HDL-C ratio that underlies AIP [33–35]. Additionally, antioxidants and phytochemicals in plant foods reduce systemic inflammation and improve insulin sensitivity, both of which suppress hepatic VLDL production and decrease circulating triglycerides [19,36]. The typically lower intake of saturated fat and dietary cholesterol in vegan diets further promotes a more favorable lipid profile. These mechanistic pathways are consistent with previous evidence; for example, a cross-sectional study found that high adherence to plant-based and healthy plant-based diets was associated with reduced odds of high-risk AIP [37]. Similarly, triglyceride and HDL levels were significantly more favorable in the high vegan adherence group in our study, aligning with prospective data from Romania, where plant-based dietary adherence normalized most lipid parameters within one year except for HDL [38]. Furthermore, anthropometric indices—including BMI, waist, hip, and wrist circumferences—were significantly lower in the high adherence group, consistent with previous reports [39,40].

The cardiovascular benefits of plant-based diets have been consistently demonstrated in previous studies [41–43]. In our study, ASCVD scores were slightly lower among individuals with high adherence to the vegan pattern, although the differences did not reach statistical significance. Nonetheless, this trend is generally in line with earlier findings indicating that healthier dietary patterns are associated with a reduced risk of cardiovascular disease [42,44]. For instance, Ghasempour Dabaghi et al. reported a 23% lower likelihood of premature coronary artery disease among those with the highest adherence to a healthy diet compared with those in the lowest adherence group [45]. Moreover, in the present study, higher adherence to the vegan dietary pattern was inversely associated with the prevalence of Metabolic Syndrome, defined by the presence of abdominal obesity, elevated triglycerides, low HDL cholesterol, high blood pressure, or fasting blood glucose ≥100 mg/dL or physician-diagnosed diabetes, which is consistent with the broader literature [22].

In the Western dietary pattern, there were no significant differences in AIP between adherence groups. This may suggest that the presence of unhealthy fats, refined carbohydrates, animal protein, salt, and additives in low amounts provides no advantage over high amounts in terms of balancing pro-atherogenic (TG) and anti-atherogenic (HDL-C) lipids or the development of atherogenic plaque. However, it was observed that participants with high adherence to the Western diet had marginally higher LDL-C levels compared to other adherence groups. In contrast to our findings, a previous study reported significant differences in all lipid profiles, including total cholesterol, LDL-C, HDL-C, and TG, among different adherence groups within the Western diet [45]. Moreover, given that smokers had significantly higher AIP than non-smokers, exploratory analysis suggested a borderline smoking × Western adherence interaction, with differences in AIP between high and low Western adherence being smaller among smokers, warranting further study. There is a proven association between the Western diet and anthropometric measures, including BMI, WC, and WrC [46,47]. Similarly, our findings suggest that high adherence to the Western diet is linked to increased values in these measurements [46,47]. ASCVD scores tended to be higher in participants with high adherence to the Western dietary pattern, indicating a modest potential impact of this dietary pattern on cardiovascular risk. This trend aligns with prior studies showing that lower adherence to a Western diet is associated with reduced prevalence of MetS [22]. In total, given the established link between Western dietary patterns and cardiovascular disease, these findings may still indicate a potential long-term risk [48–50].

Total cholesterol and LDL-C were significantly higher, and HDL-C was significantly lower in the high carbohydrate adherence groups compared to other groups, but TG, AIP, and ASCVD risk score did not differ among the groups. This is consistent with findings in the gibel carp study, where a high-carbohydrate, low lipid diet (HCLL) led to increased triglyceride levels, lipid accumulation, and enhanced lipogenesis [51]. In a systematic review and meta-analysis of overweight or obese T2DM patients, the low-carbohydrate diet significantly improved the lipid profile by reducing triglycerides and increasing HDL-C, without affecting LDL-C or total cholesterol [52]. Another meta-analysis found that low-carbohydrate diets improved cardiovascular risk factors by reducing triglycerides and increasing HDL cholesterol, without affecting LDL cholesterol or total cholesterol. However, there was little to no difference in changes to cardiovascular risk factors between low-carbohydrate and balanced-carbohydrate diets over both short-term and long-term periods, similar to the present study [53]. These results underscore the complex interplay between dietary carbohydrates, lipid metabolism, genetic factors, and probable cardiovascular disease risk. FBS levels did not differ among carbohydrate adherence groups, but participants with a history of diabetes were significantly more prevalent in the high adherence group. This suggests a potential association between higher carbohydrate adherence and a history of diabetes [54], although the direct relationship with FBS levels remains unclear and requires further investigation. Low adherence to a carbohydrate-rich diet was associated with a lower BMI, which aligns with previous studies suggesting that low-carbohydrate diets promote weight loss primarily through water and glycogen loss [52,55,56]. Moreover, for the high-carbohydrate pattern, interactions with BMI indicated that differences in AIP across adherence groups were smaller at higher BMI, suggesting that higher BMI may attenuate the impact of dietary adherence on atherogenic risk. However, the long-term effects of such diets remain uncertain [57].

This study’s strength lies in its large sample size and the application of statistical methods to identify dietary patterns, offering valuable insights into real-world dietary behaviors and their association with cardiovascular risk factors. However, its cross-sectional design limits causal inference, and the reliance on self-reported data from food frequency questionnaires may introduce bias. Additionally, the study did not account for potential confounders such as physical activity or genetic factors. It is a single-center study, and its findings may not be generalizable to other populations. Despite these limitations, the study provides important information on the link between dietary patterns and AIP as a novel predictor of cardiovascular events.

## Conclusion

This study provides valuable insights into the relationship between dietary patterns, AIP, and CVD risk factors among adults in the study population. The findings demonstrate that higher AIP is associated with a higher ASCVD risk score. High adherence to a vegan diet may have the most beneficial effects on AIP compared to other adherence patterns and diets. Furthermore, this dietary pattern is associated with lower cardiovascular risk, as evidenced by improved lipid profiles (lower TG, higher HDL-C), reduced BMI, WC, HC, and SBP. In contrast, high adherence to the Western diet is associated with higher BMI, WC, SBP, and ASCVD risk score, while high adherence to the Carbohydrate diet is linked to higher BMI, HC, TC, HDL-C, and LDL-C compared to intermediate and lower adherence groups. However, the differences in AIP with respect to atherosclerosis for the Western and Carbohydrate diets are less pronounced. While the study’s cross-sectional design limits the ability to draw causal conclusions, the results underscore the importance of dietary patterns and their potential impact on AIP as a promising biomarker for early cardiovascular risk assessment. Given the high burden of CVD in the population, these findings emphasize the need for public health initiatives targeting dietary improvements to reduce cardiovascular risk.

## Supporting information

S1 Table Mediation analysis of HDL on ASCVD risk through AIP showing direct, indirect, total effects, and proportion mediated. ACME (Average Causal Mediation Effect) represents the indirect effect of HDL on ASCVD risk operating through AIP. ADE (Average Direct Effect) represents the effect of HDL on ASCVD risk through all other pathways not involving AIP. The Total Effect is the sum of the direct and indirect effects. The Proportion Mediated is the ratio ACME/Total Effect.
(DOCX)

S2 Table Association of dietary pattern adherence with AIP in the total population and sensitivity subgroups. Data are presented as mean ± SD for AIP values and as β coefficients with standard errors (SE) and 95% confidence intervals (CI) from adjusted regression models. All models were adjusted for age, sex, and smoking.
(DOCX)

S3 Table Interaction analyses of dietary pattern adherence with age, sex, BMI, and smoking in relation to AIP. Data are presented as β coefficients with standard errors (SE) and 95% confidence intervals (CI) from regression models including main effects and interaction terms. All models were adjusted for age, sex, and smoking.
(DOCX)

S1 Fig Mediation analysis of the association between HDL and 10-year ASCVD risk through the AIP. The diagram illustrates both the direct pathway (ADE, Average Direct Effect) from HDL to ASCVD risk and the indirect pathway (ACME, Average Causal Mediation Effect) through AIP. The total effect is the sum of direct and indirect effects. HDL, high-density lipoprotein cholesterol; AIP, atherogenic index of plasma; ASCVD, atherosclerotic cardiovascular disease; ACME, average causal mediation effect (indirect effect); ADE, average direct effect; CI, confidence interval.
(PNG)

## Abbreviations

AIP: Atherogenic Index of Plasma
BMI: Body Mass Index
CI: Confidence Interval
CVD: Cardiovascular disease
DBP: Diastolic Blood Pressure
DM: Diabetes Mellitus
FBS: Fasting Blood Sugar
FFQ: Food Frequency Questionnaire
HC: Hip Circumference
HDL-C: High-Density Lipoprotein Cholesterol
HLP: Hyperlipidemia
HTN: Hypertension
LDL-C: Low-Density Lipoprotein Cholesterol
MetS: Metabolic Syndrome
PCA: Principal Component Analysis
SD: Standard Deviation
SBP: Systolic Blood Pressure
TG: Triglycerides
WHR: Waist-to-Hip Ratio
WC: Waist Circumference
WrC: Wrist Circumference
