# Supplementary material for: Association between dietary pattern, atherogenic index of plasma, and cardiovascular disease risk factors amongst adults: A cross-sectional cohort-based study
Source: PLoS One. 2026 Feb 26;21(2):e0343023. doi: 10.1371/journal.pone.0343023 (PMC12944721; doi:10.1371/journal.pone.0343023)
Supplement: S1 Table — ACME (Average Causal Mediation Effect) represents the indirect effect of HDL on ASCVD risk operating through AIP. ADE (Average Direct Effect) represents the effect of HDL on ASCVD risk through all other pathways not involving AIP. The Total Effect is the sum of the direct and indirect effects. The Proportion Mediated is the ratio ACME/Total Effect. (DOCX) [file pone.0343023.s001.docx]

**S1 Table:** Mediation analysis of HDL on ASCVD risk through AIP showing direct, indirect, total effects, and proportion mediated.

| **Effect Type** | **Estimate (β)** | **95% CI** | **p-value** |
| --- | --- | --- | --- |
| **ACME (Indirect Effect)** | -0.066 | -0.091, -0.041 | <0.001 |
| **ADE (Direct Effect)** | -0.094 | -0.135, -0.055 | <0.001 |
| **Total Effect** | -0.16 | -0.194, -0.129 | <0.001 |
| **Proportion Mediated** | 0.413 | 0.251, 0.604 | <0.001 |

ACME (Average Causal Mediation Effect) represents the indirect effect of HDL on ASCVD risk operating through AIP. ADE (Average Direct Effect) represents the effect of HDL on ASCVD risk through all other pathways not involving AIP. The Total Effect is the sum of the direct and indirect effects. The Proportion Mediated is the ratio ACME/Total Effect.
