# Supplementary material for: Association between dietary pattern, atherogenic index of plasma, and cardiovascular disease risk factors amongst adults: A cross-sectional cohort-based study
Source: PLoS One. 2026 Feb 26;21(2):e0343023. doi: 10.1371/journal.pone.0343023 (PMC12944721; doi:10.1371/journal.pone.0343023)
Supplement: S2 Table — Data are presented as mean ± SD for AIP values and as β coefficients with standard errors (SE) and 95% confidence intervals (CI) from adjusted regression models. All models were adjusted for age, sex, and smoking. (DOCX) [file pone.0343023.s002.docx]

**S2 Table:** Association of Dietary Pattern Adherence with AIP in the Total Population and Sensitivity Subgroups

| **Population  Group (n)** | **Diet** | **Adherence  Level** | **n** | **AIP (mean ± SD)** | **Comparison** | **β (Estimate)** | **SE** | **95% CI** | **p-value** |
| --- | --- | --- | --- | --- | --- | --- | --- | --- | --- |
| **Total (1675)** | **Vegan** | Low | 559 | 0.46 ± 0.23 | — | — | — |  | — |
|  |  | Medium | 554 | 0.46 ± 0.22 | Medium vs Low | -0.0041 | 0.013 | [-0.0296, 0.0214] | 0.757 |
|  |  | High | 562 | 0.41 ± 0.22 | High vs Low | **-0.04657** | **0.013** | **[-0.0721, -0.0211]** | **<0.001** |
|  | **Western** | Low | 561 | 0.45 ± 0.23 | — | — | — |  | — |
|  |  | Medium | 554 | 0.44 ± 0.22 | Medium vs Low | -0.00172 | 0.0136 | [-0.0284, 0.0249] | 0.899 |
|  |  | High | 560 | 0.45 ± 0.22 | High vs Low | 0.02159 | 0.014 | [-0.0059, 0.0490] | 0.123 |
|  | **High-carb** | Low | 553 | 0.44 ± 0.22 | — | — | — |  | — |
|  |  | Medium | 563 | 0.45 ± 0.22 | Medium vs Low | 0.01153 | 0.0137 | [-0.0153, 0.0384] | 0.399 |
|  |  | High | 559 | 0.46 ± 0.23 | High vs Low | -0.00159 | 0.0141 | [-0.0292, 0.0261] | 0.91 |
| **No HTN (1304)** | **Vegan** | Low | 418 | 0.456 ± 0.236 | — | — | — |  | — |
|  |  | Medium | 431 | 0.462 ± 0.232 | Medium vs Low | 0.00072 | 0.0155 | [-0.0297, 0.0311] | 0.963 |
|  |  | High | 455 | 0.414 ± 0.226 | High vs Low | **-0.04103** | **0.0153** | **[-0.0710, -0.0110]** | **0.007** |
|  | **Western** | Low | 451 | 0.451 ± 0.240 | — | — | — |  | — |
|  |  | Medium | 422 | 0.431 ± 0.227 | Medium vs Low | -0.00396 | 0.0155 | [-0.0343, 0.0264] | 0.799 |
|  |  | High | 431 | 0.446 ± 0.228 | High vs Low | 0.01973 | 0.0159 | [-0.0114, 0.0509] | 0.214 |
|  | **High-carb** | Low | 449 | 0.459 ± 0.241 | — | — | — |  | — |
|  |  | Medium | 435 | 0.449 ± 0.232 | Medium vs Low | 0.01484 | 0.0155 | [-0.0155, 0.0452] | 0.34 |
|  |  | High | 420 | 0.421 ± 0.220 | High vs Low | -0.00242 | 0.0162 | [-0.0342, 0.0293] | 0.881 |
| **No DM (1349)** | **Vegan** | Low | 445 | 0.459 ± 0.233 | — | — | — |  | — |
|  |  | Medium | 444 | 0.452 ± 0.222 | Medium vs Low | -0.00941 | 0.015 | [-0.0388, 0.0199] | 0.532 |
|  |  | High | 460 | 0.403 ± 0.233 | **High vs Low** | **-0.05354** | **0.0149** | **[-0.0827, -0.0243]** | **<0.001** |
|  | **Western** | Low | 476 | 0.438 ± 0.240 | — | — | — |  | — |
|  |  | Medium | 461 | 0.437 ± 0.228 | Medium vs Low | 0.01553 | 0.01492 | [-0.0137, 0.0448] | 0.298 |
|  |  | High | 412 | 0.438 ± 0.223 | High vs Low | 0.02289 | 0.01567 | [-0.0078, 0.0536] | 0.144 |
|  | **High-carb** | Low | 482 | 0.452 ± 0.237 | — | — | — |  | — |
|  |  | Medium | 445 | 0.440 ± 0.225 | Medium vs Low | 0.00824 | 0.0151 | [-0.0214, 0.0378] | 0.585 |
|  |  | High | 422 | 0.418 ± 0.228 | High vs Low | -0.0035 | 0.0157 | [-0.0343, 0.0273] | 0.824 |
| **No HLP (1075)** | **Vegan** | Low | 357 | 0.443 ± 0.235 | — | — | — |  | — |
|  |  | Medium | 353 | 0.431 ± 0.213 | Medium vs Low | -0.0104 | 0.01654 | [-0.0428, 0.0220] | 0.529 |
|  |  | High | 365 | 0.383 ± 0.227 | **High vs Low** | **-0.05722** | **0.0164** | **[-0.0894, -0.0251]** | **<0.001** |
|  | **Western** | Low | 377 | 0.430 ± 0.237 | — | — | — |  | — |
|  |  | Medium | 361 | 0.410 ± 0.221 | Medium vs Low | -0.00589 | 0.01657 | [-0.0384, 0.0266] | 0.722 |
|  |  | High | 337 | 0.415 ± 0.221 | High vs Low | 0.00555 | 0.01726 | [-0.0283, 0.0394] | 0.748 |
|  | **High-carb** | Low | 404 | 0.440 ± 0.237 | — | — | — |  | — |
|  |  | Medium | 350 | 0.421 ± 0.214 | Medium vs Low | -0.0029 | 0.01641 | [-0.0351, 0.0293] | 0.86 |
|  |  | High | 321 | 0.389 ± 0.223 | High vs Low | -0.02785 | 0.01728 | [-0.0617, 0.0060] | 0.107 |

Data are presented as mean ± SD for AIP values and as β coefficients with standard errors (SE) and 95% confidence intervals (CI) from adjusted regression models. All models were adjusted for age, sex, and smoking.
