# Supplementary material for: Association between dietary pattern, atherogenic index of plasma, and cardiovascular disease risk factors amongst adults: A cross-sectional cohort-based study
Source: PLoS One. 2026 Feb 26;21(2):e0343023. doi: 10.1371/journal.pone.0343023 (PMC12944721; doi:10.1371/journal.pone.0343023)
Supplement: S3 Table — Data are presented as β coefficients with standard errors (SE) and 95% confidence intervals (CI) from regression models including main effects and interaction terms. All models were adjusted for age, sex, and smoking. (DOCX) [file pone.0343023.s003.docx]

**S3 Table: Interaction Analyses of Dietary Pattern Adherence with Age, Sex, BMI, and Smoking in Relation to AIP**

| **Diet** | **Interaction** | **Comparison (Adherence)** | **β (Estimate)** | **Std. Error** | **95% CI** | **p-value** |
| --- | --- | --- | --- | --- | --- | --- |
| **Vegan** | **Age** | Medium vs Low | 0.00055 | 0.00164 | [-0.00266, 0.00376] | 0.736 |
|  |  | High vs Low | -0.00136 | 0.00163 | [-0.00455, 0.00183] | 0.403 |
|  | **Sex** | Medium vs Low | -0.02292 | 0.02694 | [-0.07570, 0.02990] | 0.395 |
|  |  | High vs Low | 0.01717 | 0.02702 | [-0.03580, 0.07010] | 0.525 |
|  | **BMI** | Medium vs Low | -0.00448 | 0.00301 | [-0.01038, 0.00142] | 0.137 |
|  |  | High vs Low | -0.00213 | 0.00277 | [-0.00756, 0.00330] | 0.444 |
|  | **Smoke** | Medium vs Low | 0.01515 | 0.0333 | [-0.05010, 0.08040] | 0.649 |
|  |  | High vs Low | -0.02473 | 0.03341 | [-0.0902, 0.0407] | 0.459 |
| **Western** | **Age** | Medium vs Low | 0.00148 | 0.00165 | [-0.00175, 0.00471] | 0.368 |
|  |  | High vs Low | -0.0006 | 0.00167 | [-0.00387, 0.00267] | 0.719 |
|  | **Sex** | Medium vs Low | -0.00178 | 0.027 | [-0.0547, 0.0511] | 0.947 |
|  |  | High vs Low | 0.05107 | 0.02781 | [-0.0035, 0.1056] | 0.066 |
|  | **BMI** | Medium vs Low | -0.00082 | 0.00294 | [-0.00658, 0.00494] | 0.781 |
|  |  | High vs Low | -0.00512 | 0.00282 | [-0.01064, 0.00040] | 0.070 |
|  | **Smoke** | Medium vs Low | -0.01856 | 0.03324 | [-0.0837, 0.0466] | 0.577 |
|  |  | High vs Low | -0.06527 | 0.03274 | [-0.1294, -0.0011] | **0.046** |
| **High-carb** | **Age** | Medium vs Low | -0.0006 | 0.00164 | [-0.00381, 0.00261] | 0.716 |
|  |  | High vs Low | 0.00011 | 0.00165 | [-0.00312, 0.00334] | 0.946 |
|  | **Sex** | Medium vs Low | 0.01043 | 0.02721 | [-0.0429, 0.0638] | 0.701 |
|  |  | High vs Low | 0.00654 | 0.02787 | [-0.0481, 0.0612] | 0.814 |
|  | **BMI** | Medium vs Low | -0.00793 | 0.00284 | [-0.01349, -0.00237] | **0.005** |
|  |  | High vs Low | -0.00771 | 0.00294 | [-0.01347, -0.00195] | **0.009** |
|  | **Smoke** | Medium vs Low | 0.02441 | 0.03193 | [-0.0382, 0.0870] | 0.445 |
|  |  | High vs Low | 0.00817 | 0.03591 | [-0.0622, 0.0786] | 0.820 |

**Data are presented as β coefficients with standard errors (SE) and 95% confidence intervals (CI) from regression models including main effects and interaction terms. All models were adjusted for age, sex, and smoking.**
