# Supplementary figures and images for: Association between dietary pattern, atherogenic index of plasma, and cardiovascular disease risk factors amongst adults: A cross-sectional cohort-based study
Source: PLoS One. 2026 Feb 26;21(2):e0343023. doi: 10.1371/journal.pone.0343023 (PMC12944721; doi:10.1371/journal.pone.0343023)

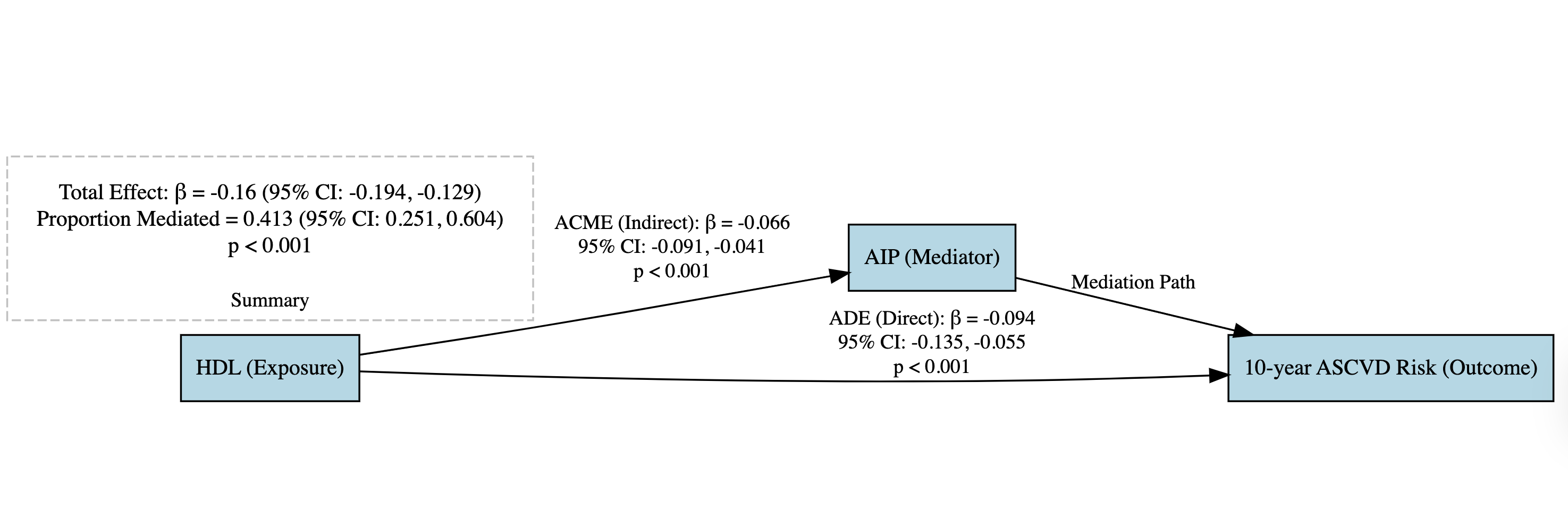

Supplement: S1 Fig — The diagram illustrates both the direct pathway (ADE, Average Direct Effect) from HDL to ASCVD risk and the indirect pathway (ACME, Average Causal Mediation Effect) through AIP. The total effect is the sum of direct and indirect effects. HDL, high-density lipoprotein cholesterol; AIP, atherogenic index of plasma; ASCVD, atherosclerotic cardiovascular disease; ACME, average causal mediation effect (indirect effect); ADE, average direct effect; CI, confidence interval. (PNG) [file pone.0343023.s004.png]
